# Impact of Anesthesia and Euthanasia on Metabolomics of Mammalian Tissues: Studies in a C57BL/6J Mouse Model

**DOI:** 10.1371/journal.pone.0117232

**Published:** 2015-02-06

**Authors:** Katherine A. Overmyer, Chanisa Thonusin, Nathan R. Qi, Charles F. Burant, Charles R. Evans

**Affiliations:** 1 Department of Molecular and Integrative Physiology, University of Michigan, Ann Arbor, Michigan, United States of America; 2 Department of Internal Medicine, University of Michigan, Ann Arbor, Michigan, United States of America; Merck & Co., UNITED STATES

## Abstract

A critical application of metabolomics is the evaluation of tissues, which are often the primary sites of metabolic dysregulation in disease. Laboratory rodents have been widely used for metabolomics studies involving tissues due to their facile handing, genetic manipulability and similarity to most aspects of human metabolism. However, the necessary step of administration of anesthesia in preparation for tissue sampling is not often given careful consideration, in spite of its potential for causing alterations in the metabolome. We examined, for the first time using untargeted and targeted metabolomics, the effect of several commonly used methods of anesthesia and euthanasia for collection of skeletal muscle, liver, heart, adipose and serum of C57BL/6J mice. The data revealed dramatic, tissue-specific impacts of tissue collection strategy. Among many differences observed, post-euthanasia samples showed elevated levels of glucose 6-phosphate and other glycolytic intermediates in skeletal muscle. In heart and liver, multiple nucleotide and purine degradation metabolites accumulated in tissues of euthanized compared to anesthetized animals. Adipose tissue was comparatively less affected by collection strategy, although accumulation of lactate and succinate in euthanized animals was observed in all tissues. Among methods of tissue collection performed pre-euthanasia, ketamine showed more variability compared to isoflurane and pentobarbital. Isoflurane induced elevated liver aspartate but allowed more rapid initiation of tissue collection. Based on these findings, we present a more optimal collection strategy mammalian tissues and recommend that rodent tissues intended for metabolomics studies be collected under anesthesia rather than post-euthanasia.

## Introduction

Samples from mammalian tissues are a prime target for metabolomics studies as they represent the sites of cellular metabolism that are disrupted in many disease states [1–4]. When properly performed, metabolomic profiling of a tissue produces a “snapshot” of its instantaneous state of metabolism and allows alterations due to disease or other perturbations to be assessed at a systems level. Rodents are a particularly useful model for study of metabolic processes in tissues because they can be easily handled and genetically manipulated in a laboratory environment, yet they maintain close similarity to humans in most aspects of cellular and systemic metabolism. Rodents can also be used to model complex human diseases such as diabetes and cardiovascular disease [5–7].

It is widely understood that proper sample preparation is a key step in metabolomics and adequate care must be taken to ensure sample fidelity lest results become laden with artifacts, confounding biological interpretation of the data [8–10]. Recent literature is replete with investigations of methodology for tissue metabolomics [11–19]. Most of these studies have focused on sample extraction and instrumental analysis methods, which are essential steps in obtaining an accurate profile of the metabolome of any biological tissue. However, the fidelity of the metabolite profile also hinges on factors determined even before metabolite extraction begins, particularly during the critical phase of tissue collection. In addition to possible trauma caused by surgical isolation, the necessary step of administering anesthetic and/or euthanizing experimental animals prior to tissue collection has substantial potential to induce changes in the metabolome.

The effects of anesthesia on metabolism have been studied previously and have been the topic of several reviews [20–22]. Most investigations pertain to the effects of longer-term (~1 hour or more) anesthetic exposure, with the goal of characterizing and managing the harmful and beneficial effects of anesthesia during surgery. Other studies have focused on understanding the impact of short-term anesthesia or euthanasia as a step in tissue collection procedures for rodents [23–28]. However, all of these reports have measured only a small number of metabolites by biochemical or other highly targeted approaches. As metabolomics has emerged as a more powerful and comprehensive means of studying the dynamics of metabolism in tissues, relatively little consideration has been given to the unique sensitivity of the metabolome to perturbation during tissue collection. This is evidenced by the fact that many recent studies do not specify the method of anesthesia used for tissue collection, or cite procedures in which rodent tissue is collected after euthanasia has been performed [14,29–32]. To assess the significance of anesthesia and tissue collection strategy for metabolomics, an in-depth comparison of different modes of anesthesia or euthanasia for the purpose of metabolomics of tissues is warranted.

In this study, we systematically examined the effect of several commonly-used methods of anesthesia and euthanasia on the metabolome of tissues in C57BL/6J mice. We employed both untargeted and targeted profiling of polar metabolites using hydrophilic interaction chromatography – electrospray ionization mass spectrometry (HILIC-ESI-MS) to assess alterations in the metabolome of skeletal muscle, heart, liver, adipose and serum. The results suggest a path to produce a more accurate and reliable portrait of tissue metabolism.

## Experimental Methods

### Materials and reagents

Carbon-13 stable isotope internal standards were purchased from Sigma-Aldrich (St. Louis, MO), Cambridge Isotope (Andover, MA), and Omicron Biochemicals (South Bend, IN). All other metabolite standards and other reagents were purchased from Sigma-Aldrich. A certified anesthetic delivery machine stocked with isoflurane was rented from the University of Michigan Unit for Laboratory Animal Medicine. Ketamine HCl (100 mg/mL) USP and sodium pentobarbital (50 mg/mL) USP were purchased from the University of Michigan Hospital Pharmacy.

### Ethics statement

All procedures involving animals in this study were carried out in accordance with the recommendations set forth in the Guide for the Care and Use of Laboratory Animals of the National Institutes of Health. The protocol was reviewed and approved by the University Committee on Use and Care of Animals (UCUCA) of the University of Michigan, Ann Arbor, protocol #PRO00003797. Surgical tissue isolations were performed as terminal procedures under anesthesia as described below, and all precautions were taken to minimize suffering.

### Animal handling

Male C57BL/6J mice, aged 20 weeks, weighing 26.8 ± 2.2 g (mean ± SD), were purchased from Jackson Laboratories (Bar Harbor, ME). The mice were maintained on a 12 h/12 h light/dark cycle and were provided with standard chow and water access ad libitum. On the day tissue collection was to be performed, mice were fasted beginning at 9 AM for a period of 5 hours, and tissue collection procedures were initiated at 2 PM.

### Tissue collection

Six different methods of anesthesia or euthanasia were evaluated for tissue collection (n = 8 mice per method of anesthesia). In all cases, tissue collection procedures were initiated after animals had either been euthanized or were under deep anesthesia and unresponsive to all stimuli. The three methods of euthanasia were: 1) cervical dislocation: mice were removed from their cages and gently restrained while resting on the benchtop. Cervical dislocation was performed manually and resulted in euthanasia within approximately 10 seconds. 2) Carbon dioxide: euthanasia was performed by introduction of 100% carbon dioxide into a bedding-free cage initially containing room air with the lid closed at a rate sufficient to induce rapid anesthesia, with death occurring within 2.5 minutes. 3) Isoflurane overdose: mice were placed into a chamber filled with vapor of the anesthetic isoflurane until respiration ceased (within 2 minutes). The three methods of anesthesia were: 4) Continuous isoflurane: using a calibrated anesthetic delivery machine, mice were induced into anesthesia at a dose of 4% isoflurane, then maintained at a surgical plane by continuous inhalation of 2% isoflurane. Typical time to initiation of tissue collection was 1.5 minutes. 5) Ketamine: a 100 mg/mL solution of ketamine in sterile saline was administered intraperitoneally (IP) at a dose of 120 mg/kg. Time to initiation of tissue collection was 20 minutes. 6) Pentobarbital: a 50 mg/mL solution of pentobarbital in sterile saline was administered IP at a dose of 60 mg/kg. Time to initiation of tissue collection was 15 minutes. Once anesthesia was induced or euthanasia was performed, collection of tissues was initiated immediately and was performed following the same procedure and timing regardless of anesthesia or euthanasia method. Tissues and blood were rapidly collected in the following order: gastrocnemius muscle, arterial blood (approximately 600 μL collected from descending aorta using a 25-gauge needle), liver, heart, and epididymal white adipose tissue. All tissues were rapidly rinsed in deionized water to remove excess blood, blotted dry and frozen by immersion in liquid nitrogen within 10 seconds of removal from the animal. Collection of all tissues was complete within 3 minutes. To prepare serum, blood was allowed to clot on ice for 15 minutes and was then centrifuged for 15 minutes at 3000 x g. The supernatant was withdrawn and frozen in liquid nitrogen. All samples were stored at -80°C until extraction.

### Tissue extraction

Frozen gastrocnemius skeletal muscle, liver, heart and epididymal white adipose tissue samples from C57BL/6J mice were ground to a homogenous powder using a liquid nitrogen-chilled mortar and pestle. Approximately 30 mg (wet mass) of each pulverized tissue sample was rapidly transferred to a pre-weighed, dry-ice-chilled 1.5 mL microcentrifuge tube, which was returned to dry ice until addition of extraction solvent. The extraction solvent consisted of a single-phase mixture of 7 parts methanol: 2 parts water: 1 part chloroform, and contained ^13^C-labeled internal standards at the concentrations specified in S1 Table. To extract the samples, 1 mL of chilled (4°C) extraction solvent was added to the tube containing the frozen pulverized tissue, after which the contents were immediately homogenized by 20 seconds of pulsed sonication using a probe sonicator (Branson 450, output power setting 4, 40% duty cycle), with the tube in an ice bath. Samples were allowed to rest 5 minutes on ice, and were then centrifuged 10 minutes at 16,000 x g at 4°C. The supernatant was withdrawn and transferred to autosampler vials for LC-MS analysis. The residual tissue pellet was dried by vacuum centrifugation and its mass was measured for normalization.

### Blood serum extraction

Serum was extracted according to a procedure described by Bruce et al. [33]. 50 μL of serum were extracted by addition of 200 μL of ice-cold 1:1:1 methanol:acetonitrile:acetone containing a mixture of ^13^C internal standards (S1 Table), followed by vigorous vortexing for 20 seconds. The samples were allowed to rest on ice for 5 minutes, and were then centrifuged for 10 minutes at 16,000 x g at 4°C. The supernatant was transferred directly to autosampler vials for LC-MS analysis.

### LC-MS methods

Samples were analyzed by LC-MS using an Agilent 1200 LC system with an Agilent 6220 time-of-flight mass spectrometer. The chromatographic method was a modified version of the mixed-mode HILIC-anion exchange separation developed by Bajad et al. [34]. Briefly, the method employs a Phenomenex (Torrance, CA) Luna 3μ NH_2_ column, 2.1 x 150 mm. Mobile phase A was acetonitrile and mobile phase B was 5mM ammonium acetate adjusted to pH 9.9 using ammonium hydroxide. The gradient was as follows: linear from 20 to 100% B over 15 minutes, 3 minute hold at 100% B, then return to 20% B at 18.1 minutes and re-equilibrate for 12 minutes. The total run time was 30 minutes and the flow rate was 0.25 mL/minute. The column temperature was 25°C and sample vials were held at 4°C in the autosampler. The injection volume was 25 μL. Mass spectrometry was performed using electrospray ionization in negative ion mode with a dual spray source with Reference mass correction enabled. Full-scan mass spectra were acquired over the m/z range 50–1200 Da with a data acquisition rate of 1 scan / second. Source parameters were: drying gas temperature 350°C, drying gas flow rate 10 L/min, nebulizer pressure 30 psig, capillary voltage 3500 V. All samples from a given tissue type were extracted and analyzed on the same day, in randomized order. A pooled sample derived from the tissue extracts was injected every ninth run throughout the analysis to monitor peak area reproducibility.

### Data analysis

Untargeted metabolite screening was performed using the metabolomics data analysis software package MZmine version 2.10 and the web-server based data processing package MetaboAnalyst version 2.0 [35,36]. Raw LC-MS data files were converted from Agilent .d format to mzXML format using Agilent MassHunter Qualitative Analysis (version B.04.00) and were then imported into MZmine. Mass detection was performed using the centroid mass detector with the noise level set at 1.0E3. The chromatogram builder was then used to generate peaks with a minimum time span of 0.2 min, minimum height of 1.0E3, an m/z tolerance of 0.002 m/z or 20 ppm. Chromatograms were smoothed with a filter width of 5. Chromatogram deconvolution was performed using the noise amplitude algorithm with a minimum peak height of 5.0E3, a peak duration range of 0–25 minutes, and 2.0E3 as the amplitude of noise setting. Isotopic peaks were grouped using an m/z tolerance of 0.002 m/z or 20 ppm, a retention time tolerance of 0.1 minutes (absolute), a maximum charge of 2, and the representative isotope set as most intense. Retention time normalization was performed with an m/z tolerance of 0.002 m/z or 20 ppm, a retention time tolerance of 1.0 min (absolute), and a minimum standard intensity of 1.0E4. Chromatograms were then aligned into a peak list using the join aligner with an m/z tolerance of 0.005 m/z or 50.0 ppm, a weight for m/z of 50, a retention time tolerance of 1.5 min (absolute) and a weight for RT of 50. Gap-filling was performed using the peak finder algorithm with an intensity tolerance of 25%, a m/z tolerance of 0.002 m/z or 20 ppm, a retention time tolerance of 1.0 min (absolute) and RT correction enabled. A duplicate peak filter was applied to remove peaks within an m/z tolerance of 0.01 m/z or 50.0 ppm, and an RT tolerance of 0.5 min (absolute). Subsequently, a peak list rows filter was applied to keep only peaks appearing in 75% of all samples, with 1 peak minimum per isotope pattern, m/z range set automatically, retention time range 1.0–25.0 minutes, and peak duration range of 0.1–2.0 minutes. The resulting peak list was then displayed as a table within MZmine and the peak shapes of all features were visually inspected, and artifact features were discarded. The data were then exported to MetaboAnalyst for multivariate statistical analysis. Data were uploaded as a peak intensity table, with missing data points handled according to default parameters. Data were filtered by interquartile range and were then normalized by median intensity and log-transformed. Principal component analysis (PCA) and partial least squares discriminant analysis (PLS-DA) were then performed. The PLS-DA model was validated by random permutation of the Y variable and by evaluating the goodness of fit (R2Y and Q2). For random permutation tests, 100 models were calculated and the goodness of fit was compared with the original model in a validation plot. Two-dimensional score plots were generated to visually assess separation between sample groups.

Targeted metabolite analysis was performed using Agilent MassHunter Quantitative Analysis software (version 4.0). Metabolite identification was accomplished by comparison of accurate mass and retention time with that of authentic standards analyzed using the same method. For relative quantitation, each metabolite was quantitated by peak area. For absolute quantitation of selected metabolites, peak areas were measured relative to the peak areas of ^13^C-labeled internal standards added to the extraction solvent. Six-point calibration curves for these compounds were generated using non-labeled authentic standard solutions spiked with the same concentrations of ^13^C-labeled internal standards as in the extraction solvent. Visual network mapping of detected metabolites was performed using Metscape 2.0 [37]. For comparisons between CD and other modes of anesthesia and euthanasia, a p-value was calculated using Student’s t-test and corrected for multiple comparisons by false discovery rate correction [38].

## Results and Discussion

**Fig 1:**
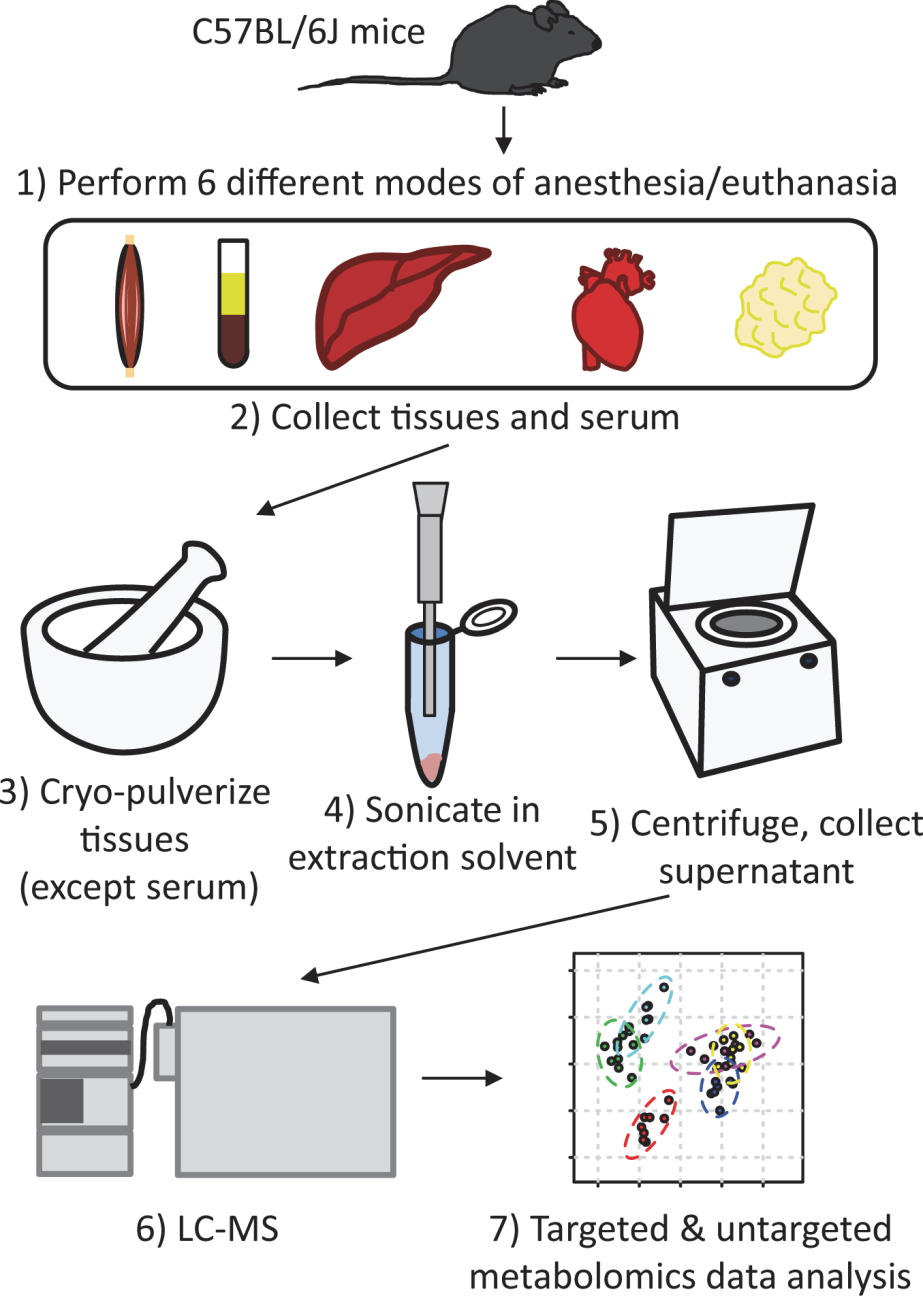
Methods workflow. C57BL/6J mice were anesthetized or euthanized using six different standard laboratory methods as described in the text. Skeletal muscle, serum, liver, heart and adipose tissue were then rapidly collected and frozen by immersion in liquid nitrogen. Tissue samples were pulverized under liquid nitrogen, then sonicated in extraction solvent. Samples were centrifuged and the supernatant was analyzed by LC-MS. Targeted and untargeted metabolomics were used to identify metabolites which differed in abundance between methods of anesthesia or euthanasia.

Several factors associated with anesthesia or euthanasia may induce alterations in the tissue metabolome. First, the absence of respiration and blood circulation in euthanized animals can induce changes in tissue metabolism compared to anesthetized, oxygenated animals. Secondly, the anesthetic or euthanasia-inducing agent may have direct effects on metabolism, despite intact circulation and respiration. Additionally, stress associated with the induction of anesthesia and surgical tissue isolation may also contribute to alterations in metabolite levels. To systematically assess the extent of variation in the metabolome induced by these factors, we examined the metabolome of skeletal muscle, heart, liver, white adipose, and serum of C57BL/6J mice immediately following three commonly-used methods of anesthesia and three methods of euthanasia, with n = 8 animals per condition, as illustrated in Fig. 1.

### Untargeted Metabolomics

**Fig 2:**
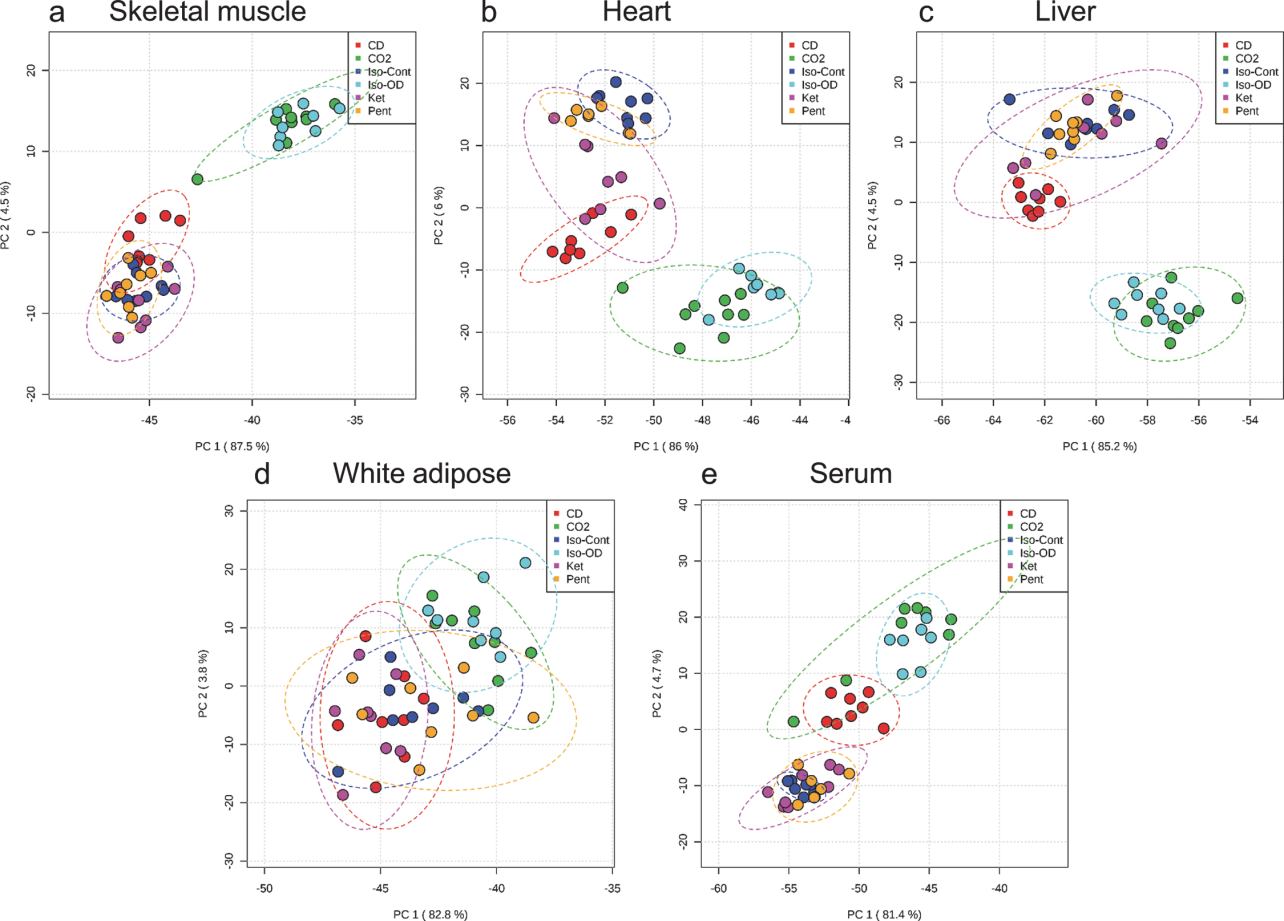
Principal component analysis of untargeted metabolomics data from tissues collected using different methods of anesthesia. Two-dimensional PCA score plots reveal separation in metabolite profiles induced by different methods of anesthesia and euthanasia in C57BL/6J mice. Tissues analyzed were a) skeletal muscle, b) heart, c) liver, d) white adipose and e) serum. Methods of anesthesia and euthanasia were: CD, cervical dislocation euthanasia (red); CO2, Carbon dioxide euthanasia (green); Iso-Cont, continuous isoflurane anesthesia (dark blue); Iso-OD, isoflurane overdose euthanasia (light blue); Ket, ketamine anesthesia (pink); Pent, pentobarbital anesthesia (orange). Ellipses represent the 95% confidence interval.

Untargeted analysis of the tissue and serum samples revealed divergent effects of different methods of anesthesia or euthanasia on the tissue metabolome. Using MetaboAnalyst 2.0, unsupervised principle component analysis (PCA) was used to generate the two-dimensional score plots shown in Fig. 2 [36]. The three methods of anesthesia, continuous inhaled isoflurane (Iso-Cont), IP ketamine (Ket) and IP pentobarbital (Pent), have mostly-overlapping distributions of data points in the score plots for all tissues. This suggests that the metabolome of tissues collected under anesthesia, in which respiration and blood circulation remain intact until the time of collection, are largely similar regardless of what anesthetic agent is used. However, the size of the ellipse representing the 95% confidence interval was larger for Ket than Iso-Cont or Pent in heart and liver, indicating ketamine may generate more variability in the metabolome. Two methods of euthanasia, inhaled isoflurane overdose (Iso-OD) and inhaled carbon dioxide (CO_2_), overlap with one another but are separated from the other methods in all tissues except for white adipose. The effect of cervical dislocation (CD), the third method of euthanasia, varied between tissues. In liver and white adipose, CD’s effects on the metabolome fall within the distribution of the three methods of anesthesia. In skeletal muscle, heart, and serum, CD appears approximately mid-way between the two other methods of euthanasia and the three methods of anesthesia. This suggests that CD induces some alterations within the metabolome compared to anesthesia, but the overall profile remains more similar to anesthetized animals, presumably due to the rapidity of CD compared to other methods of euthanasia.

**Fig 3:**
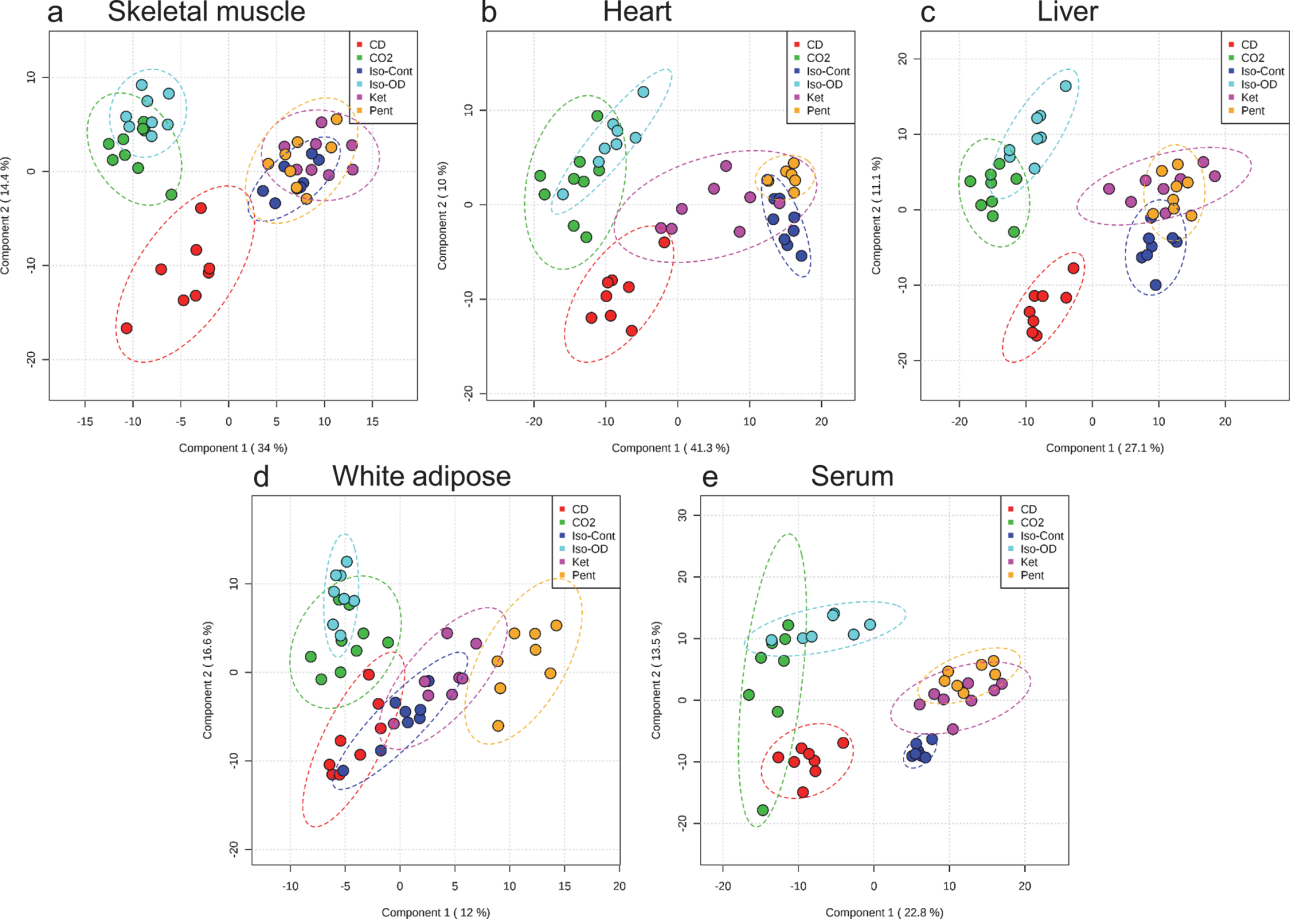
Partial least squares discriminant analysis of untargeted metabolomics data from tissues collected using different methods of anesthesia. Two-dimensional PLS-DA score plots reveal separation in metabolite profiles induced by different methods of anesthesia and euthanasia in C57BL/6J mice. Tissues analyzed were a) skeletal muscle, b) heart, c) liver, d) white adipose and e) serum. Methods of anesthesia and euthanasia were: CD, cervical dislocation euthanasia (red); CO2, Carbon dioxide euthanasia (green); Iso-Cont, continuous isoflurane anesthesia (dark blue); Iso-OD, isoflurane overdose euthanasia (light blue); Ket, ketamine anesthesia (pink); Pent, pentobarbital anesthesia (orange). Ellipses represent the 95% confidence interval.

To more specifically model differences between the methods of anesthesia or euthanasia and to select features of interest in the data, partial least squares-discriminant analysis (PLS-DA) was used [36,39]. Each model was validated by permutation tests based on separation distance as described by Rubingh et al [40], which resulted in a between/within (B/W) ratio outside the distribution of random class assignments (p-value <0.01) for all tissues. Fig. 3 contains the 2D score plots for the PLS-DA classification models. The distinctions observed between methods of anesthesia and methods of euthanasia are mainly similar to the results from PCA, although the separation between groups is cleaner, as is typical for a supervised data transformation methods [41]. Notably, in skeletal muscle, heart and liver, CD is separated from all other methods to a greater extent than was observed in the PCA score plots, and this separation is mainly along the secondary component axis.

To investigate which metabolites were most responsible for the observed differences between methods of anesthesia or euthanasia, features from each tissue were ranked by variable importance in projection (VIP) scores based on the PLS-DA classification model [36]; features with higher VIP scores contribute to a greater degree to the class separation. The m/z values of the features in these ranked lists were searched against the online Human Metabolome Database (HMDB) [42] with a mass accuracy of 20 ppm, and the top 10 from each tissue with putative database matches are listed as annotated features in Table 1. The table contains compounds from a variety of metabolite classes, including glycolysis and TCA cycle intermediates, lipids and nucleosides. The lists differ substantially between tissues, suggesting that each tissue metabolome has a distinct response to different anesthesia or euthanasia methods. Several of the highest-ranking annotated features in skeletal muscle are glycolytic metabolites, as well as phosphocreatinine and phosphocreatine. Lipid species are more prominent in the ranked metabolite lists for liver and adipose tissue. Some metabolites in the table are in common between tissues, including succinic acid (skeletal muscle, heart, adipose), glycerol 3-phosphate (skeletal muscle, heart and serum), inosine monophosphate (heart, liver and adipose) and ceramide phosphates (skeletal muscle, heart and adipose). This suggests that these metabolites may be more universal hallmarks of response to euthanasia or specific anesthetics. However, the metabolite names listed in Table 1 can only be considered putative identifications, and many highly-ranked features were disregarded due to the absence of database matches. We therefore opted to proceed with further analysis using a targeted approach.

**Table 1:** Annotated features most responsible for differentiating methods of anesthesia and euthanasia in each tissue as determined by PLS-DA analysis.

	Putative HMDB database match	Observed m/z	Retention time (min)
Skeletal muscle			
	Succinic acid	117.018	13.97
	Glycerol 3-phosphate	171.005	14.6
	Hexose phosphate	259.021	14.77
	Hexose bisphosphate	338.998	14.75
	D-erythrose 4-phosphate	199.000	14.74
	Glutaconic acid	129.018	2.1166
	Acetylphosphate	138.978	14.76
	Phosphocreatinine	192.017	13.80
	Phosphocreatine	210.027	13.80
	Ceramide phosphate (18:1/16:0)	616.462	2.75
Heart			
	13S-hydroxyoctadecadienoic acid	295.222	4.43
	Uridine triphosphate	482.96	20.56
	Inosine monophosphate	347.039	16.73
	Ceramide phosphate (18:0/16:0)	618.475	2.79
	6-phosphogluconic acid	275.02	14.38
	Succinic acid	117.018	14.16
	Glycerol 3-phosphate	171.005	14.53
	Propionic acid	73.029	14.17
	Adenosine triphosphate	505.989	20.74
	Phosphocreatine	210.027	13.990
Liver			
	5-L-glutamyl-taurine	253.054	10.64
	Inosine monophosphate	347.039	16.66
	Uracil	111.019	3.68
	3-Sulfinoalanine	152.001	8.10
	Eicosapentaenoic acid	301.216	4.87
	Eicosenoic acid	309.278	4.22
	D-pantothenoyl-L-cysteine	357.088	14.4
	Quinolinic acid	166.016	7.97
	Pentadecanoic acid	241.216	4.91
	α-ketoglutaric acid	145.013	14.78
Adipose			
	3-oxooctadecanoic acid	297.242	5.49
	LysoPE (16:1)	450.266	3.42
	7Z,10Z-Hexadecadienoic acid	251.200	5.10
	Isobutyrylglycine	144.065	8.07
	Palmitoleic acid	253.216	5.04
	Ceramide phosphate (18:0/16:0)	618.476	2.77
	Inosine monophosphate	347.039	17.96
	Citrulline	174.085	7.26
	Phosphatidylglycerol (18:1/18:1)	773.533	2.84
	Succinic acid	117.018	15.02
Serum			
	Hypoxanthine	135.03	6.34
	Acetylcarnitine	202.107	6.37
	D-Ribose-5-phosphate	229.01	13.98
	Xanthine	187.005	3.72
	5-HETE	319.226	4.13
	Glycerol-3-phosphate	171.006	13.95	
	Isovalerylalanine	172.097	6.10
	13-OxoODE	293.209	4.03
	α-ketoisovaleric acid	115.04	5.95
	D-glucuronic acid	193.036	10.68

### Targeted Metabolomics

**Fig 4:**
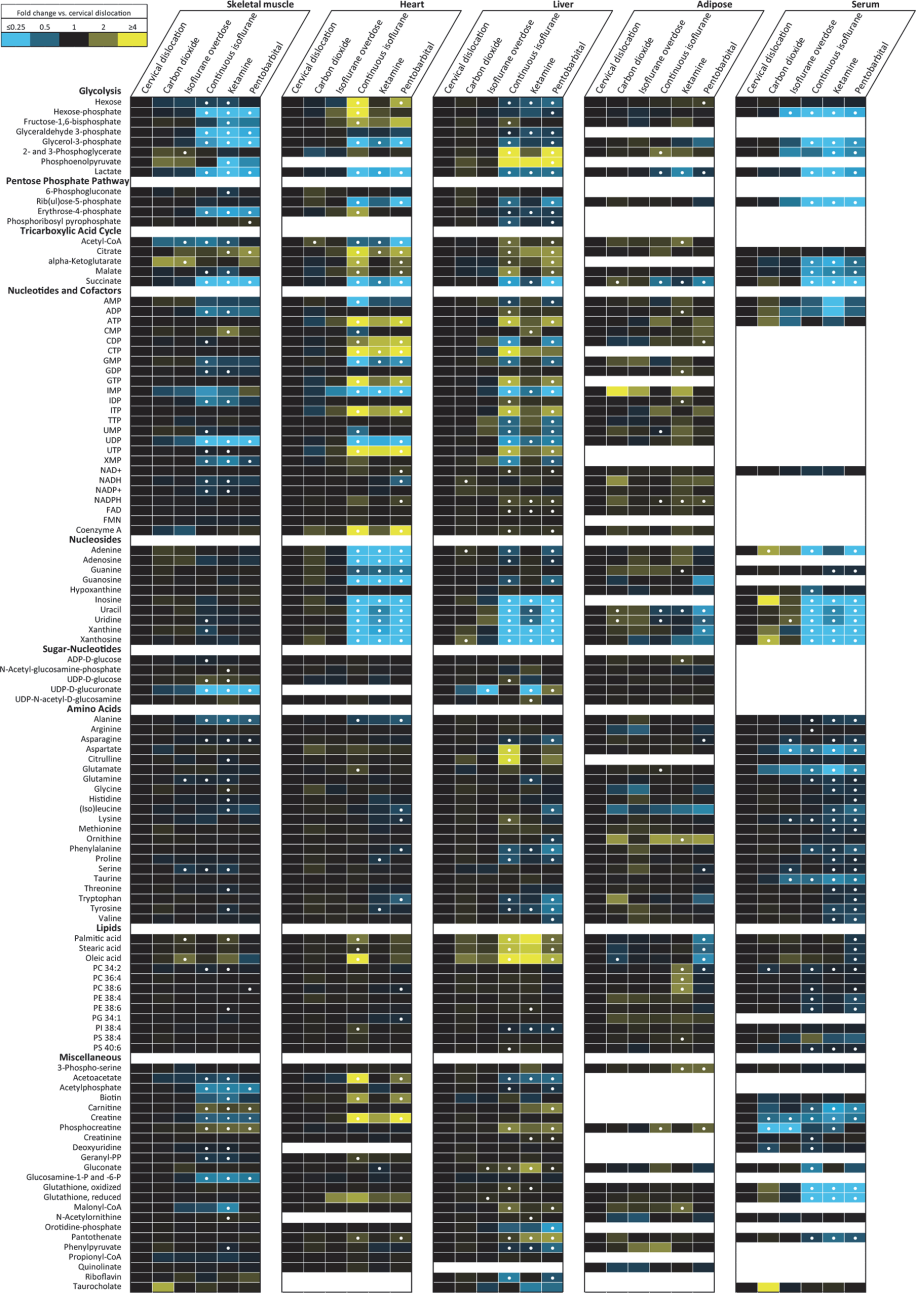
Heatmap illustrating alterations in metabolite levels in tissues collected using different methods of anesthesia. Data are expressed as fold change versus cervical dislocation. n = 8 mice per method of anesthesia or euthanasia. • indicates p < 0.05 versus cervical dislocation after false discovery rate correction.

In order to more fully elucidate the effect of anesthesia or euthanasia methods on known metabolic pathways, 112 known polar metabolites, many of which are intermediates or cofactors in central carbon metabolism, were quantitated by peak area. These compounds have been previously analyzed as authentic standards on our HILIC LC-MS platform and are identified using both accurate mass and retention time, as listed in S2 Table. Of these compounds, absolute concentrations could be obtained for 21 metabolites because matching ^13^C internal standards were included in the extraction solvent. Our measurements for these metabolites in the tissue extracts are found in S3 Table. For all 112 metabolites, relative quantitation by peak area was used to assess differences in metabolite levels between different methods of anesthesia or euthanasia. Fig. 4 is a heatmap that illustrates the fold-change of each metabolite relative to its abundance in tissues collected using cervical dislocation. Differences were considered significant for p ≤ 0.05 after false discovery rate correction [38]. Cervical dislocation was selected as the reference method as it does not involve administration of any exogenous chemical anesthetics. The most apparent trend in the targeted profiles is the distinction between animals euthanized prior to tissue collection (CD, CO_2_ and Iso-OD) and the methods in which the tissues were collected under anesthesia (Iso-Cont, Ket and Pent). Although the mechanism for this distinction cannot be definitively assigned using the data from this study, the data are consistent with the effects of hypoxia brought about by the absence of respiration and blood circulation in euthanized animals. Detailed analysis of the effects of anesthesia and euthanasia on selected metabolic pathways is provided below.

**Fig 5:**
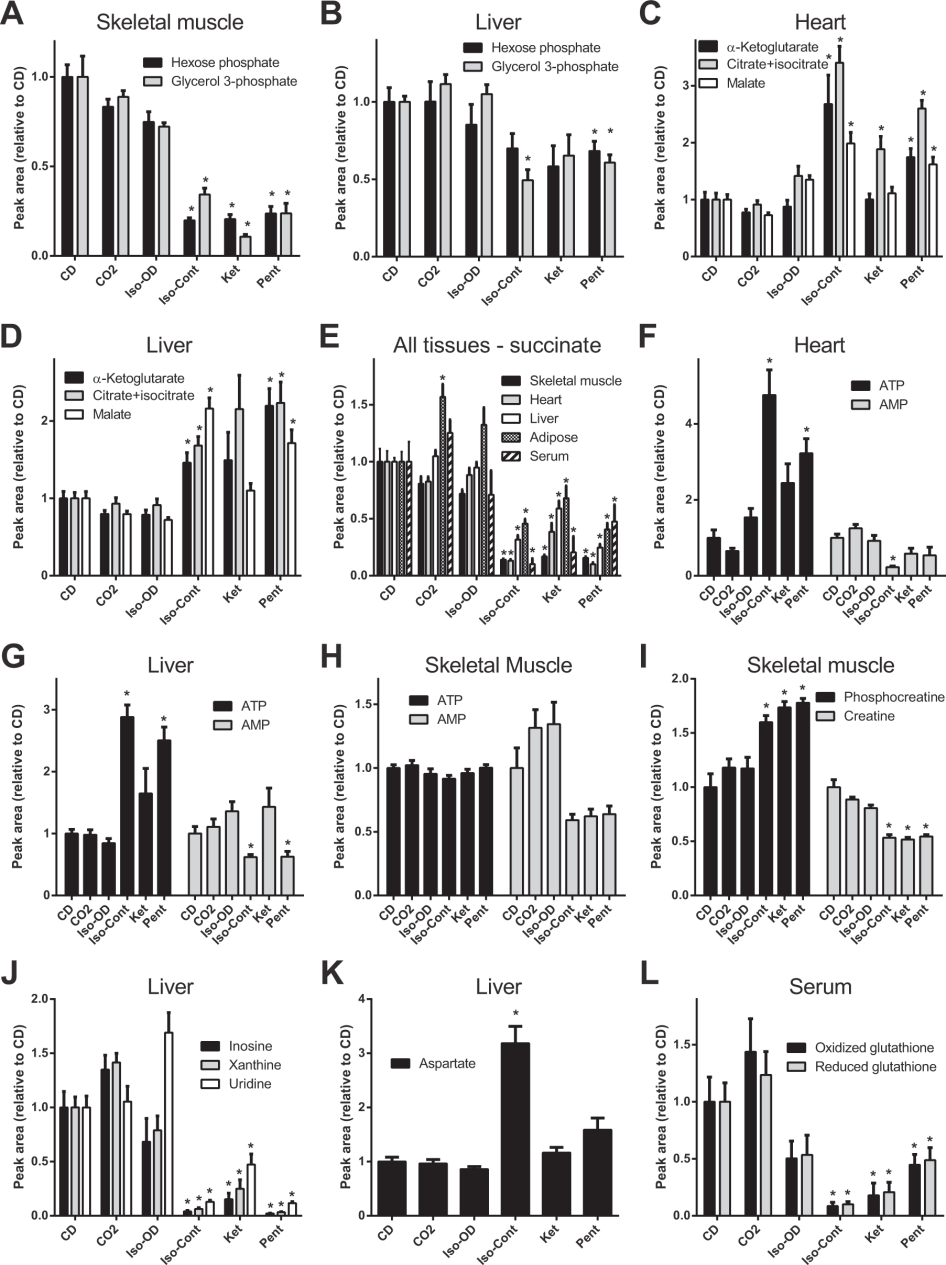
Impact of anesthesia or euthanasia on selected metabolites. Data are presented as mean ± standard error of the mean and expressed as peak area relative to cervical dislocation (CD) for each method of anesthesia or euthanasia. n = 8 mice per method of euthanasia. * indicates p < 0.05 versus cervical dislocation after false discovery rate correction.

**Glycolysis and glycogenolysis.** Higher lactate levels were observed in all tissues and in serum when tissue was collected from euthanized animals compared to collection under all methods of anesthesia. This is consistent with a switch from oxidative to non-oxidative carbohydrate metabolism due to reduced availability of oxygen, which results in activation of glycolysis to compensate for reduced energy production through oxidative phosphorylation. In skeletal muscle, metabolites associated with glycolysis, including hexose phosphates and glycerol 3-phosphate, were also higher in euthanized animals (Fig. 5A), likely reflecting increased mobilization of glycogen. Alterations of hexose phosphate and glycerol-3-phosphate were observed to a lesser extent in liver (Fig. 5B), but not in cardiac muscle or adipose tissue, where glycogen stores are less abundant or absent. Hexose phosphates and other phosphorylated glycolysis intermediates are not considered to be substantial components of the serum metabolome, but nonetheless these species were detected at significantly elevated levels in serum of euthanized animals (Fig. 4), suggesting that short-term hypoxia may cause enough tissue damage to release significant amounts of intracellular metabolites into the blood.

**TCA cycle.** In heart and liver, citrate, α-ketoglutarate and malate levels were lower in euthanized mice compared to animals under isoflurane or pentobarbital-induced anesthesia, suggesting cataplerotic consumption of these TCA intermediates (Fig. 5C, 5D). In contrast, succinate levels were higher in all tissues of euthanized mice compared to anesthetized mice (Fig. 5E). Elevation of succinate in mammalian tissue is a well-documented response to hypoxia [43,44]. This phenomenon was observed even in the first tissue collected, gastrocnemius muscle, which was frozen less than one minute following euthanasia. This underscores the rapidity of the response of the tissue metabolome to loss of blood circulation. In contrast to the tissue, serum levels of α-ketoglutarate and malate were elevated in euthanized animals (Fig. 4), providing possible evidence for increased secretion of these metabolites, and/or their release from damaged cells following euthanasia.

**Nucleotides and nucleosides.** In heart and liver of euthanized mice there were 4.8 and 2.9-fold lower levels of ATP, respectively, compared to anesthetized animals, while levels of AMP were higher (Fig. 5F, 5G). In skeletal muscle ATP was much less affected by method of anesthesia or euthanasia (Fig. 5H). AMP in skeletal muscle was elevated slightly, although its absolute abundance is more than 300-fold lower than ATP (S3 Table). This maintenance of ATP in skeletal muscle likely is attributed to the presence of the phosphocreatine-creatine buffer system, which can anaerobically donate phosphate to ADP to maintain ATP levels. This buffer system is important in skeletal muscle during periods of high ATP demand such as intense bursts of contraction, but it also apparently acts to maintain ATP during the brief period of hypoxia which precedes tissue collection in euthanatized animals, as well as during the contractions in leg muscles that occur immediately following cervical dislocation. Correspondingly, phosphocreatine was partially depleted (1.6-fold, p = 0.001) and creatine increased (1.9-fold, p <0.001) in the skeletal muscle of euthanized animals (Fig. 5I).

In the liver, heart and serum, metabolites involved in purine metabolism – which include the nucleosides inosine, xanthine, uridine and several others – were all markedly higher in euthanized animals than anesthetized animals (Figs. 4 and 5J). A metabolic network representing these species is illustrated in S1 Fig. The elevation of nucleosides may be caused by an increase in breakdown of mono and di-nucleotides such as ADP and AMP, as a mechanism of disposing of excess nucleotide species. The absence of accumulation of these species in skeletal muscle under similar hypoxia is explained by the maintenance of ATP levels by phosphocreatine as already described. It is also noteworthy that most nucleotides in the liver of mice that were administered ketamine did not differ from mice euthanized by cervical dislocation, unlike mice anesthetized using continuous isoflurane and pentobarbital. Liver is a major site of metabolism of ketamine, and these results are consistent with a previous study suggesting that ketamine induces alterations in energy metabolism in liver compared to the inhaled anesthetic isoflurane. [45]. Notably, ketamine was slower and more variable in time to induction of surgical-plane anesthesia, which may contribute to the increased variability in levels of some metabolites. Adipose tissue nucleotide levels were generally less affected by method of anesthesia than muscle or liver (Fig. 4). Serum of euthanized mice contained increased levels of circulating nucleosides compared to anesthetized mice, possibly due to export of these compounds into circulation by the liver or due to the result of tissue damage from hypoxia (Fig. 4).

**Lipids.** In liver, fatty acids including palmitic acid, stearic acid and oleic acid were lower in liver of euthanized animals. In other tissues, specific responses to individual anesthetics were observed. Pentobarbital resulted in reduced levels of free fatty acids in adipose tissue, while ketamine caused elevated levels of phosphatidylcholine (PC) species (Fig. 4).

**Other metabolites.** In all tissues, amino acids, electron transfer metabolites NAD(P) and NADP(H) and sugar-nucleotides except UDP-glucuronate were mostly unaffected or showed relatively small (<1.5) fold-change versus cervical dislocation. A noteworthy exception was elevated levels of aspartate in liver of animals administered continuous isoflurane compared to other methods of anesthesia (3-fold higher vs. CD and 1.9-fold higher vs. pentobarbital, p<0.01, Fig. 5K). Although isoflurane has been reported to alter liver metabolism [45], the cause of the acute change in aspartate levels is unknown. Additionally, the abundant antioxidant metabolite glutathione, which exists in both reduced and oxidized forms, was unaffected by method of anesthesia or euthanasia in tissues but was significantly higher in serum of euthanized animals (oxidized form 10-fold higher for cervical dislocation vs. continuous isoflurane, p = 0.005, Fig. 5L), providing further evidence for leakage of damaged cells after euthanasia.

### Comparison with Prior Studies

Our results are generally consistent with and substantially extend previous literature describing the effect of anesthesia and/or euthanasia on rodent tissue metabolism. Brooks and colleagues examined the effects of euthanasia on rat liver, comparing euthanasia by decapitation, CO_2_, CO_2_/O_2_, and isoflurane-induced euthanasia [25]. Their results showed evidence of accumulation of selected glycolytic metabolites and increased breakdown of glycogen in liver following isoflurane or CO_2_-induced euthanasia as compared to decapitation. These authors also noted that surgical-plane isoflurane anesthesia caused smaller, statistically non-significant alterations in glycogen utilization and ATP production, which agrees well with our findings. In another work, Faupel et al. studied the effect of euthanasia and anesthesia on the concentrations of 18 nucleotide, TCA and glycolysis metabolites in rat liver [46]. Their data indicated that cervical dislocation induced elevation of AMP, in agreement with our findings. Their study also compared pentobarbital anesthesia to a rapid method of mechanical euthanasia using a custom-designed double hatchet apparatus. Although effective in preventing muscle contractions and reducing the hypoxic period prior to tissue sampling, the apparatus required considerable operator skill and effectively limited rapid collection of non-hypoxic tissue to organs found within the abdominal cavity.

### Recommended Strategies for Tissue Metabolomics

Limitations in tissue collection speed are a potential issue when multiple organs are to be harvested for metabolomics. In our own work, this likely accentuated the differences observed in metabolite levels between euthanized and anesthetized animals, as the hypoxic period was longer for tissues which were collected later in the procedure. Much of this concern can be eliminated by collecting tissues under anesthesia, but it should nevertheless be standard practice for the organ or tissue of highest priority for metabolomic analysis to be collected first, with other tissues collected immediately thereafter in a consistent order and at consistent time intervals.

Others have recommended alternative tissue collection strategies for metabolomics, such as perfusing liver with saline solution prior to freezing [47]. This method has the advantage of removing the majority of entrained blood, allowing intracellular liver metabolites to be distinguished from metabolites originating from residual blood within the tissue. However, the procedure takes approximately 1 minute and induces modest tissue damage. As indicated by our study, the response of the metabolome to cessation of blood circulation is rapid, and the intracellular metabolome cannot be expected to remain stable during this period. It is therefore our preference to capture the combined tissue and residual blood metabolome from anesthetized animals, as this is likely to more closely reflect true in vivo conditions.

Our study was limited to C57BL/6J mice. Other species may respond differently to anesthesia or euthanasia, although it is likely that many of the metabolic responses observed are shared by other mammals. We have carried out limited studies on the effect of anesthesia and euthanasia on the metabolome of rat tissues (S2 Fig.). In this experiment, isoflurane anesthesia was compared to euthanasia by guillotine, which is a more standard procedure than cervical dislocation in larger rodents. Mirroring trends in mice, euthanized rats had higher levels of glycolytic intermediates and succinate in skeletal muscle and liver, as well as higher liver AMP and reduced liver ATP. As in mice, these observations are compatible with tissue response to hypoxia following euthanasia.

Some principals from our study may also be translated to preparation of human biopsy samples for metabolomics, which is an area of growing interest in cancer research and other fields [48–50]. The procedure for biopsy in humans is necessarily less flexible than tissue sampling in rodents, as minimizing risk of injury to the subject is paramount. Biopsies are always carried out under local, regional or general anesthesia with blood circulation intact, thus there is less concern of tissue hypoxia prior to and during sampling. Direct effects of anesthetics on metabolism, as we observed in rodents, are likely to occur and will depend on the class of anesthetic and the tissue being biopsied. Another critical step is tissue handling immediately after biopsy. Typical histological applications require formalin fixed or, less frequently, fresh frozen samples for sectioning. For the purpose of metabolomics, immediate freezing of tissue at liquid-nitrogen temperature with minimal manipulation is strongly preferred, as our data in rodents show that even one minute without blood circulation is sufficient to induce significant alterations in the metabolome. Brown et al. demonstrated immersion of tissue in 80% methanol, without tissue disruption, as an alternative procedure for both tissue fixation and metabolite extraction, although quantitative reproducibility was not assessed [48]. Some classes of metabolites may be stable against more harsh procedures, as recent work has shown that even archival formalin-fixed, paraffin-embedded tissue may be of some utility for metabolomics [50]. Regardless of the procedure which is chosen, consistency in timing and execution of the biopsy and tissue handling procedure is essential in order to minimize disruption of the metabolome.

## Conclusions

Taken together, the data from the present work and other studies suggest that hypoxia induced by euthanasia causes rapid and dramatic alterations in many portions of the C57BL/6J mouse metabolome. We thus recommend that tissues be collected from living, anesthetized animals, following all ethical standards and after full institutional animal use committee review and approval. Each method of anesthesia also induces specific changes in the metabolome, which although smaller than the drastic changes caused by euthanasia, may need to be considered when interpreting results. In our hands, administration of isoflurane by an anesthetic vaporizer machine was the most satisfactory method due to rapid induction and low variability in time-to-collection between animals. It should also be noted that, despite large differences between anesthesia and euthanasia, most methods produced a metabolite profile which was relatively consistent between replicates. Thus, if a specific mode of anesthesia or even euthanasia is required for other studies, it may still be possible to obtain useful metabolome profiles from collected tissues. In all cases, the procedure used for anesthesia and tissue collection should be fully documented in all publications.

## Supporting Information

S1 Fig Network view of metabolites associated with purine synthesis and degradation. Metabolites in red hexagons were detected by LC-MS; pink hexagons indicate compounds not detected that directly connect via known metabolic pathways to observed metabolites. Metabolites shown as small hexagons were less abundant in liver of mice anesthetized by isoflurane than those euthanized by cervical dislocation. Large hexagons indicate higher abundance in isoflurane-anesthetized mice. Green outlines on hexagons indicate statistical significance (p<0.05 after FDR correction). Network view was generated using Metscape 2.0. [37].
(DOCX)

S2 Fig Relative metabolite levels in tissues collected from rats immediately following euthanasia (decapitation) or under anesthesia (isoflurane). Data represent averages of biological replicates (n = 2 per condition). Rats were high capacity running females [51], aged 12 weeks, body weight 161 +/- 12 g. Tissue dissection order and timing was the same as specified for mice in the experimental methods.
(DOCX)

S1 Table Calibration parameters and internal standards used for absolute quantitation of selected metabolites in tissue extracts. Calibration curve fits were linear and were generated using the ratio of the unlabeled standard peak area to the corresponding stable-isotope internal standard peak area. Stable isotope internal standard suppliers were: ^a^ Omicron Biochemical, ^b^ Sigma-Aldrich, ^c^ Cambridge Isotope.
(DOCX)

S2 Table List of metabolites detected by targeted analysis in at least one tissue type. Metabolites are specified by common name and InChI Key identifier codes. In cases where multiple isomers are possible but are not distinguished (e.g., phospholipids such as PG_36:4), the InChI Key refers to a single representative isomer. Metabolite observed m/z values and RT values are given for metabolites detected in liver extract. Observed m/z values typically agree within +/- 10 ppm with m/z values predicted from molecular formulas.
(DOCX)

S3 Table Measured concentrations of endogenous metabolites in tissues of C57BL/6J mice. Tissues were collected after a 5 hour fast, under isoflurane anesthesia. Values are expressed as mean (standard deviation). n = 8 mice. nd = not detected; nq = below limit of quantitation.
(DOCX)
