# Supplementary material for: Impact of Anesthesia and Euthanasia on Metabolomics of Mammalian Tissues: Studies in a C57BL/6J Mouse Model
Source: PLoS One. 2015 Feb 6;10(2):e0117232. doi: 10.1371/journal.pone.0117232 (PMC4319778; doi:10.1371/journal.pone.0117232)
Supplement: S1 Fig — Metabolites in red hexagons were detected by LC-MS; pink hexagons indicate compounds not detected that directly connect via known metabolic pathways to observed metabolites. Metabolites shown as small hexagons were less abundant in liver of mice anesthetized by isoflurane than those euthanized by cervical dislocation. Large hexagons indicate higher abundance in isoflurane-anesthetized mice. Green outlines on hexagons indicate statistical significance (p<0.05 after FDR correction). Network view was generated using Metscape 2.0. [37]. (DOCX) [file pone.0117232.s001.docx]

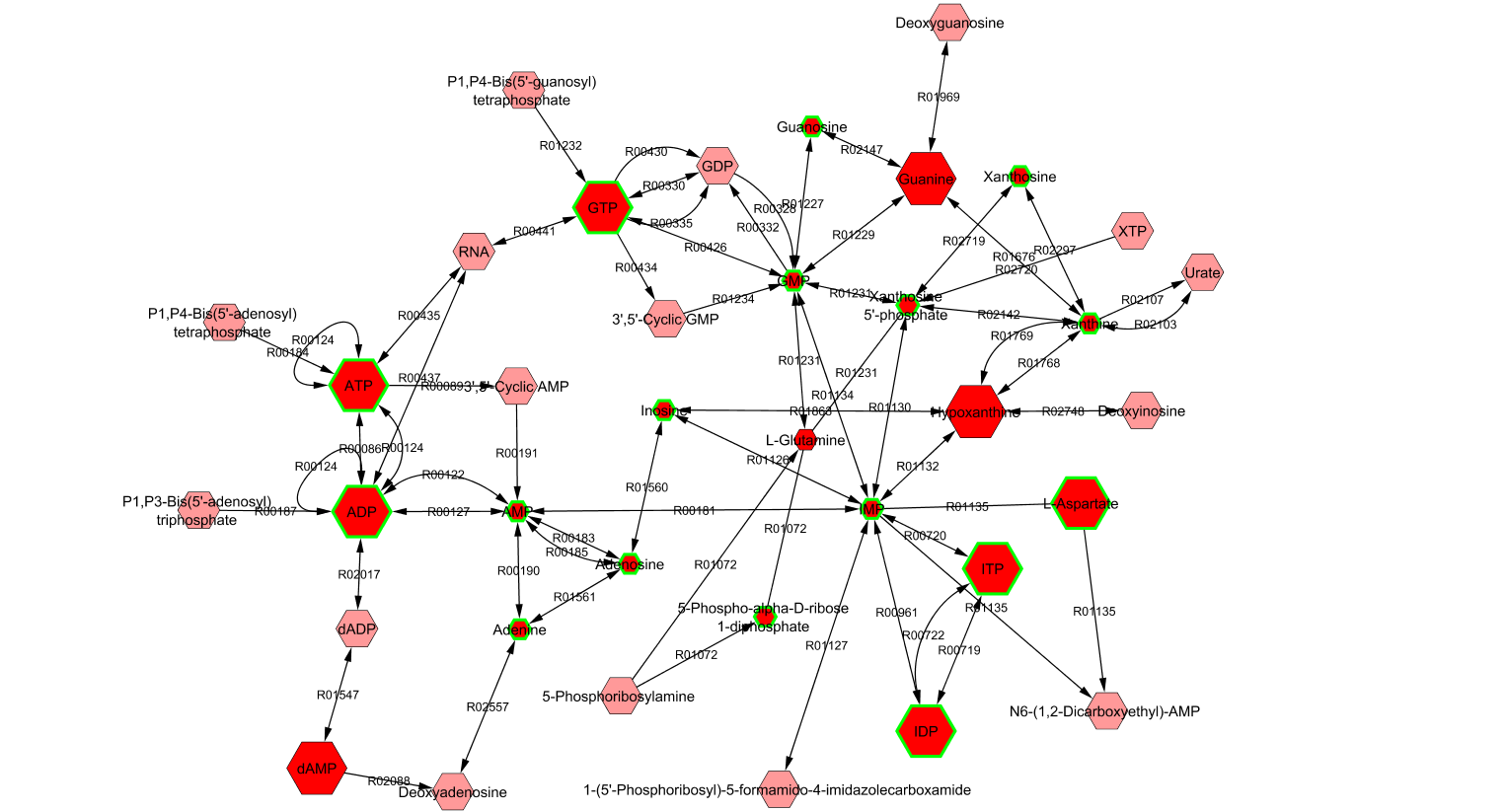


**Figure S1. Network view of metabolites associated with purine synthesis and degradation.** Metabolites in red hexagons were detected by LC-MS; pink hexagons indicate compounds not detected that directly connect via known metabolic pathways to observed metabolites. Metabolites shown as small hexagons were less abundant in liver of mice anesthetized by isoflurane than those euthanized by cervical dislocation. Large hexagons indicate higher abundance in isoflurane-anesthetized mice. Green outlines on hexagons indicate statistical significance (p<0.05 after FDR correction). Network view was generated using Metscape 2.0. [[37](#_ENREF_37)].
