# Supplementary material for: Impact of Anesthesia and Euthanasia on Metabolomics of Mammalian Tissues: Studies in a C57BL/6J Mouse Model
Source: PLoS One. 2015 Feb 6;10(2):e0117232. doi: 10.1371/journal.pone.0117232 (PMC4319778; doi:10.1371/journal.pone.0117232)
Supplement: S2 Fig — Data represent averages of biological replicates (n = 2 per condition). Rats were high capacity running females [51], aged 12 weeks, body weight 161 +/- 12 g. Tissue dissection order and timing was the same as specified for mice in the experimental methods. (DOCX) [file pone.0117232.s002.docx]

**Figure S2. Relative metabolite levels in tissues collected from rats immediately following euthanasia (decapitation) or under anesthesia (isoflurane).** Data represent averages of biological replicates (n=2 per condition). Rats were high capacity running females [[51](#_ENREF_51)], aged 12 weeks, body weight 161 +/- 12 g. Tissue dissection order and timing was the same as specified for mice in the experimental methods.
