# Supplementary material for: Impact of Anesthesia and Euthanasia on Metabolomics of Mammalian Tissues: Studies in a C57BL/6J Mouse Model
Source: PLoS One. 2015 Feb 6;10(2):e0117232. doi: 10.1371/journal.pone.0117232 (PMC4319778; doi:10.1371/journal.pone.0117232)
Supplement: S1 Table — Calibration curve fits were linear and were generated using the ratio of the unlabeled standard peak area to the corresponding stable-isotope internal standard peak area. Stable isotope internal standard suppliers were: a Omicron Biochemical, b Sigma-Aldrich, c Cambridge Isotope. (DOCX) [file pone.0117232.s003.docx]

**Table S1.** **Calibration parameters and internal standards used for absolute quantitation of selected metabolites in tissue extracts.** Calibration curve fits were linear and were generated using the ratio of the unlabeled standard peak area to the corresponding stable-isotope internal standard peak area. Stable isotope internal standard suppliers were: ^a^ Omicron Biochemical, ^b^ Sigma-Aldrich, ^c^ Cambridge Isotope.

| **Metabolite (IS isotope, supplier)** | Calibration curve concentrations (µM) | R^2^ | Internal standard concentration added to extraction solvent | | | | |
| --- | --- | --- | --- | --- | --- | --- | --- |
|  |  |  | **Skeletal muscle** | **Liver** | **Heart** | **Adipose** | **Serum** |
| Fructose 6-phosphate (^13^C_6_, ^a^) | 0, 0.5, 2, 5, 20, 100 | 0.999 | 20 µM | 4 µM | 8 µM | 2 µM | 2 µM |
| Fructose 1,6-bisphosphate (^13^C_6_, ^a^) | 0, 0.1, 0.4 ,1, 4, 20 | 0.999 | 8 µM | 1 µM | 8 µM | 0.2 µM | 0.8 µM |
| Lactate (^13^C_2_, ^b^) | 0, 2, 8, 20, 80, 400 | 1.000 | 40 µM | 40 µM | 80 µM | 20 µM | 400 µM |
| Citrate (^13^C_6_, ^c^) | 0, 0.25, 1, 2.5, 10, 50 | 0.999 | 2 µM | 0.4 µM | 8 µM | 2 µM | 20 µM |
| α-ketoglutarate (^13^C_4_, ^c^) | 0, 0.1, 0.4 ,1, 4, 20 | 0.999 | 0.8 µM | 0.2 µM | 0.4 µM | 0.2 µM | 4 µM |
| Succinate (^13^C_4_, ^c^) | 0, 0.5, 2, 5, 20, 100 | 0.999 | 4 µM | 4 µM | 20 µM | 1 µM | 40 µM |
| Malate (^13^C_4_, ^c^) | 0, 0.5, 2, 5, 20, 100 | 0.999 | 4 µM | 4 µM | 8 µM | 1 µM | 10 µM |
| Adenosine monophosphate (^13^C_10_^15^N_5,_ ^b^) | 0, 0.5, 2, 5, 20, 100 | 0.999 | 0.8 µM | 10 µM | 8 µM | 2 µM | 2 µM |
| Adenosine diphosphate (^13^C_10_^15^N_5,_ ^b^) | 0, 0.5, 2, 5, 20, 100 | 0.999 | 4 µM | 10 µM | 4 µM | 2 µM | 2 µM |
| Adenosine triphosphate (^13^C_10_^15^N_5,_ ^b^) | 0, 0.5, 2, 5, 20, 100 | 0.999 | 40 µM | 20 µM | 40 µM | 4 µM | 4 µM |
| Amino acids  (algal-derived U- ^13^C mix, ^b^) | 0, 0.25, 1, 2.5, 10, 50 | 0.998 to 1.000 | 10 µg/mL | 10 µg/mL | 20 µg/mL | 4 µg/mL | 20 µg/mL |
