# Supplementary material for: Impact of Anesthesia and Euthanasia on Metabolomics of Mammalian Tissues: Studies in a C57BL/6J Mouse Model
Source: PLoS One. 2015 Feb 6;10(2):e0117232. doi: 10.1371/journal.pone.0117232 (PMC4319778; doi:10.1371/journal.pone.0117232)
Supplement: S2 Table — Metabolites are specified by common name and InChI Key identifier codes. In cases where multiple isomers are possible but are not distinguished (e.g., phospholipids such as PG_36:4), the InChI Key refers to a single representative isomer. Metabolite observed m/z values and RT values are given for metabolites detected in liver extract. Observed m/z values typically agree within +/- 10 ppm with m/z values predicted from molecular formulas. (DOCX) [file pone.0117232.s004.docx]

**Table S2.** **List of metabolites detected by targeted analysis in at least one tissue type.** Metabolites are specified by common name and InChI Key identifier codes. In cases where multiple isomers are possible but are not distinguished (e.g., phospholipids such as PG_36:4), the InChI Key refers to a single representative isomer. Metabolite observed m/z values and RT values are given for metabolites detected in liver extract. Observed m/z values typically agree within +/- 10 ppm with m/z values predicted from molecular formulas.

| Metabolite name | Observed m/z | Observed RT (min) | InChI Key |
| --- | --- | --- | --- |
| 3-Phosphoglycerate | 184.9851 | 17.60 | OSJPPGNTCRNQQC-UHFFFAOYSA-N |
| 3-Phospho-serine | 184.0011 | 7.25 | BZQFBWGGLXLEPQ-UHFFFAOYSA-N |
| 6-Phospho-D-gluconate | 275.0168 | 16.99 | BIRSGZKFKXLSJQ-SQOUGZDYSA-N |
| Acetoacetate | 101.0239 | 4.48 | WDJHALXBUFZDSR-UHFFFAOYSA-N |
| Acetyl-CoA | 403.5556 | 19.53 | ZSLZBFCDCINBPY-ZSJPKINUSA-N |
| Acetylphosphate | 138.9796 | 14.72 | LIPOUNRJVLNBCD-UHFFFAOYSA-N |
| Adenine | 134.0467 | 3.27 | GFFGJBXGBJISGV-UHFFFAOYSA-N |
| Adenosine | 266.0889 | 3.27 | OIRDTQYFTABQOQ-KQYNXXCUSA-N |
| ADP | 426.0216 | 18.42 | XTWYTFMLZFPYCI-KQYNXXCUSA-N |
| ADP-D-glucose | 588.0744 | 15.28 | WFPZSXYXPSUOPY-ROYWQJLOSA-N |
| Alanine | 88.0399 | 5.65 | QNAYBMKLOCPYGJ-REOHCLBHSA-N |
| alpha-Ketoglutarate | 145.0137 | 14.82 | KPGXRSRHYNQIFN-UHFFFAOYSA-N |
| AMP | 346.0553 | 15.05 | UDMBCSSLTHHNCD-KQYNXXCUSA-N |
| Arginine | 173.1038 | 5.95 | ODKSFYDXXFIFQN-BYPYZUCNSA-N |
| Asparagine | 131.0457 | 8.44 | DCXYFEDJOCDNAF-REOHCLBHSA-N |
| Aspartate | 132.0297 | 11.69 | CKLJMWTZIZZHCS-REOHCLBHSA-N |
| ATP | 505.9879 | 19.48 | ZKHQWZAMYRWXGA-KQYNXXCUSA-N |
| Biotin | 243.0803 | 6.55 | YBJHBAHKTGYVGT-ZKWXMUAHSA-N |
| Carnitine | 160.0974 | 6.47 | PHIQHXFUZVPYII-ZCFIWIBFSA-N |
| CDP | 402.0104 | 14.99 | ZWIADYZPOWUWEW-XVFCMESISA-N |
| Citrate | 191.0192 | 16.59 | KRKNYBCHXYNGOX-UHFFFAOYSA-N |
| CMP | 322.0440 | 14.82 | IERHLVCPSMICTF-XVFCMESISA-N |
| Coenzyme A | 766.1074 | 19.96 | RGJOEKWQDUBAIZ-DRCCLKDXSA-N |
| Creatine | 130.0622 | 5.69 | CVSVTCORWBXHQV-UHFFFAOYSA-N |
| Creatinine | 112.0516 | 2.99 | DDRJAANPRJIHGJ-UHFFFAOYSA-N |
| CTP | 481.9767 | 19.26 | PCDQPRRSZKQHHS-XVFCMESISA-N |
| Deoxyuridine | 227.0668 | 7.94 | MXHRCPNRJAMMIM-SHYZEUOFSA-N |
| Hexose-phosphate | 259.0219 | 14.77 | NBSCHQHZLSJFNQ-GASJEMHNSA-N |
| Dihydroxy-acetone phosphate | 168.9902 | 15.45 | GNGACRATGGDKBX-UHFFFAOYSA-N |
| D-Rib(ul)ose-5-phosphate | 229.0113 | 14.74 | KTVPXOYAKDPRHY-SOOFDHNKSA-N |
| Erythrose-4-phosphate | 199.0008 | 14.77 | NGHMDNPXVRFFGS-IUYQGCFVSA-N |
| FAD | 784.1493 | 14.41 | VWWQXMAJTJZDQX-UYBVJOGSSA-N |
| FMN | 455.0968 | 14.87 | FVTCRASFADXXNN-SCRDCRAPSA-N |
| Fructose-1,6-bisphosphate | 338.9882 | 19.41 | RNBGYGVWRKECFJ-ZXXMMSQZSA-N |
| GDP | 442.0165 | 19.54 | QGWNDRXFNXRZMB-UUOKFMHZSA-N |
| Gluconate | 195.0505 | 10.21 | RGHNJXZEOKUKBD-SQOUGZDYSA-N |
| Glucosamine 6-phosphate | 258.0379 | 13.26 | XHMJOUIAFHJHBW-UKFBFLRUSA-N |
| Glutamate | 146.0453 | 11.89 | WHUUTDBJXJRKMK-VKHMYHEASA-N |
| Glutamine | 145.0613 | 7.91 | ZDXPYRJPNDTMRX-VKHMYHEASA-N |
| Glycerol-3-phosphate | 171.0058 | 14.55 | AWUCVROLDVIAJX-GSVOUGTGSA-N |
| Glycine | 74.0242 | 14.19 | DHMQDGOQFOQNFH-UHFFFAOYSA-N |
| GMP | 362.0502 | 16.25 | RQFCJASXJCIDSX-UUOKFMHZSA-N |
| GTP | 521.9828 | 20.94 | XKMLYUALXHKNFT-UUOKFMHZSA-N |
| Guanine | 150.0416 | 6.23 | UYTPUPDQBNUYGX-UHFFFAOYSA-N |
| Guanosine | 282.0838 | 6.15 | NYHBQMYGNKIUIF-UUOKFMHZSA-N |
| Histidine | 154.0616 | 8.41 | HNDVDQJCIGZPNO-YFKPBYRVSA-N |
| Hypoxanthine | 135.0307 | 9.90 | FDGQSTZJBFJUBT-UHFFFAOYSA-N |
| IDP | 427.0056 | 18.42 | XZQMKKFWMMGK-VTHZCTBJSA-N |
| IMP | 347.0393 | 16.68 | PGZVUSPTYXQADT-KQYNXXCUSA-N |
| Inosine | 267.0729 | 7.25 | UGQMRVRMYYASKQ-KQYNXXCUSA-N |
| ITP | 506.9719 | 19.46 | HAEJPQIATWHALX-KQYNXXCUSA-N |
| Lactate | 89.0240 | 9.42 | JVTAAEKCZFNVCJ-REOHCLBHSA-N |
| Leucine / Isoleucine | 130.0868 | 4.94 | ROHFNLRQFUQHCH-YFKPBYRVSA-N |
| Lysine | 145.0977 | 10.97 | KDXKERNSBIXSRK-YFKPBYRVSA-N |
| Malate | 133.0137 | 14.21 | BJEPYKJPYRNKOW-UHFFFAOYSA-N |
| Malonyl-CoA | 403.5556 | 19.53 | LTYOQGRJFJAKNA-VFLPNFFSSA-N |
| Methionine | 148.0432 | 6.22 | FFEARJCKVFRZRR-BYPYZUCNSA-N |
| Hexose | 179.0556 | 4.84 | CDAISMWEOUEBRE-GPIVLXJGSA-N |
| N-Acetyl-glucosamine-1-phosphate | 300.0484 | 14.24 | FZLJPEPAYPUMMR-RTRLPJTCSA-N |
| N-Acetylornithine | 173.0926 | 5.95 | JRLGPAXAGHMNOL-LURJTMIESA-N |
| NAD+ | 662.1013 | 11.69 | BAWFJGJZGIEFAR-NNYOXOHSSA-O |
| NADH | 664.1169 | 14.51 | BOPGDPNILDQYTO-NNYOXOHSSA-N |
| NADP+ | 742.0676 | 17.27 | XJLXINKUBYWONI-NNYOXOHSSA-O |
| NADPH | 744.0833 | 20.07 | ACFIXJIJDZMPPO-NCHANQSKSA-N |
| Oleic acid | 281.2480 | 4.54 | ZQPPMHVWECSIRJ-KTKRTIGZSA-N |
| Ornithine | 131.0820 | 10.69 | AHLPHDHHMVZTML-BYPYZUCNSA-N |
| Orotidine-phosphate | 367.0179 | 14.74 | KYOBSHFOBAOFBF-XVFCMESISA-N |
| Glutathione, oxidized | 611.1441 | 15.75 | YPZRWBKMTBYPTK-UHFFFAOYSA-N |
| Palmitic acid | 255.2330 | 4.76 | IPCSVZSSVZVIGE-UHFFFAOYSA-N |
| Pantothenate | 218.1028 | 8.85 | GHOKWGTUZJEAQD-UHFFFAOYSA-N |
| PC_34:2 | 816.5750 | 2.46 | QJZAWHQVXYMEQR-RWGOWQMXSA-N |
| PC_36:4 | 840.5760 | 2.41 | PUDHBQQGURLBGJ-IVFHWKNFSA-N |
| PC_38:6 | 864.5760 | 2.41 | ZZSZQATYGVHNLZ-PUMLCSCDSA-N |
| PE_38:4 | 766.5392 | 2.59 | YEHAZLUVAZKVRO-PDWSRYNESA-N |
| PE_38:6 | 762.5079 | 2.62 | LFGBKOUQHCWBQI-BZGLIJSBSA-N |
| PG_34:1 | 747.5170 | 3.52 | GTCKEWVHTGGUSN-HGWHEPCSSA-N |
| Phenylalanine | 164.0712 | 5.95 | COLNVLDHVKWLRT-QMMMGPOBSA-N |
| Phenylpyruvate | 163.0395 | 7.43 | BTNMPGBKDVTSJY-UHFFFAOYSA-N |
| Phosphocreatine | 210.0290 | 13.86 | DRBBFCLWYRJSJZ-UHFFFAOYSA-N |
| Phosphoenolpyruvate | 166.9746 | 18.38 | DTBNBXWJWCWCIK-UHFFFAOYSA-N |
| PI_38:4 | 885.5510 | 4.43 | CBYJKEBKBMWPMN-ITNYWZHKSA-N |
| Proline | 114.0555 | 6.20 | ONIBWKKTOPOVIA-BYPYZUCNSA-N |
| Propionyl-CoA | 822.1336 | 11.69 | QAQREVBBADEHPA-UXYNFSPESA-N |
| Phosphoribosyl pyrophosphate | 388.9440 | 14.70 | PQGCEDQWHSBAJP-TXICZTDVSA-N |
| PS_38:4 | 810.5300 | 6.68 | UTSXKUOHAAGJOV-JLUUWOCLSA-N |
| PS_40:6 | 834.5290 | 6.57 | YZPDRIODLGOXPQ-VQXQEPRVSA-N |
| Quinolinate | 166.0140 | 14.80 | GJAWHXHKYYXBSV-UHFFFAOYSA-N |
| Glutathione, reduced | 306.0760 | 13.51 | RWSXRVCMGQZWBV-WDSKDSINSA-N |
| Riboflavin | 375.1305 | 3.02 | AUNGANRZJHBGPY-SCRDCRAPSA-N |
| Serine | 104.0348 | 8.24 | MTCFGRXMJLQNBG-REOHCLBHSA-N |
| Stearic acid | 283.2640 | 4.41 | QIQXTHQIDYTFRH-UHFFFAOYSA-N |
| Succinate | 117.0188 | 14.06 | KDYFGRWQOYBRFD-UHFFFAOYSA-N |
| Taurine | 124.0068 | 7.71 | XOAAWQZATWQOTB-UHFFFAOYSA-N |
| Taurocholic acid | 514.2850 | 7.31 | WBWWGRHZICKQGZ-BKXUXKPJSA-N |
| Threonine | 118.0504 | 7.84 | AYFVYJQAPQTCCC-GBXIJSLDSA-N |
| Tryptophan | 203.0820 | 6.04 | QIVBCDIJIAJPQS-VIFPVBQESA-N |
| TTP | 480.9814 | 15.00 | NHVNXKFIZYSCEB-XLPZGREQSA-N |
| Tyrosine | 180.0661 | 7.44 | OUYCCCASQSFEME-QMMMGPOBSA-N |
| UDP | 402.9944 | 14.52 | XCCTYIAWTASOJW-XVFCMESISA-N |
| UDP-D-glucose | 565.0472 | 14.44 | HSCJRCZFDFQWRP-LPTOLDDLSA-N |
| UDP-D-glucuronate | 579.0265 | 17.27 | HDYANYHVCAPMJV-LXQIFKJMSA-N |
| UDP-N-acetyl-D-glucosamine | 606.0737 | 14.32 | LFTYTUAZOPRMMI-CFRASDGPSA-N |
| UMP | 323.0280 | 14.99 | DJJCXFVJDGTHFX-XVFCMESISA-N |
| Uracil | 111.0195 | 3.68 | ISAKRJDGNUQOIC-UHFFFAOYSA-N |
| Uridine | 243.0617 | 3.55 | DRTQHJPVMGBUCF-XVFCMESISA-N |
| UTP | 482.9607 | 19.64 | PGAVKCOVUIYSFO-XVFCMESISA-N |
| Valine | 116.0712 | 5.72 | KZSNJWFQEVHDMF-BYPYZUCNSA-N |
| Xanthine | 151.0256 | 10.86 | LRFVTYWOQMYALW-UHFFFAOYSA-N |
| Xanthosine | 283.0679 | 11.09 | UBORTCNDUKBEOP-UUOKFMHZSA-N |
| XMP | 363.0342 | 16.25 | DCTLYFZHFGENCW-UUOKFMHZSA-N |
