# Supplementary material for: Impact of Anesthesia and Euthanasia on Metabolomics of Mammalian Tissues: Studies in a C57BL/6J Mouse Model
Source: PLoS One. 2015 Feb 6;10(2):e0117232. doi: 10.1371/journal.pone.0117232 (PMC4319778; doi:10.1371/journal.pone.0117232)
Supplement: S3 Table — Tissues were collected after a 5 hour fast, under isoflurane anesthesia. Values are expressed as mean (standard deviation). n = 8 mice. nd = not detected; nq = below limit of quantitation. (DOCX) [file pone.0117232.s005.docx]

**Table S3.** **Measured concentrations of endogenous metabolites in tissues of C57BL/6J mice.**  Tissues were collected after a 5 hour fast, under isoflurane anesthesia. Values are expressed as mean (standard deviation). n = 8 mice. nd = not detected; nq = below limit of quantitation

| **Metabolite** | Concentrations measured using stable-isotope internal standards | | | | |
| --- | --- | --- | --- | --- | --- |
|  | nmol / mg dry tissue, mean (SD) | | | | µM (SD) |
|  | **Skeletal muscle** | **Liver** | **Heart** | **Adipose** | **Serum** |
| Hexose phosphates | 3.8 (0.9) | 1.8 (0.6) | 4.2 (1.5) | 0.083 (0.032) | nq |
| Fructose 1,6-bisphosphate | 1.4 (0.9) | 0.18 (0.07) | 1.3 (0.6) | nd | nd |
| Lactate | 13 (4) | 91 (47) | 12 (4) | 2.4 (0.8) | 2000 (300) |
| Citrate + Isocitrate | 0.72 (0.07) | 0.39 (0.03) | 4.4 (1.1) | 0.44 (0.31) | 180 (20) |
| α-ketoglutarate | 0.071 (0.015) | 0.048 (0.015) | 0.3 (0.16) | 0.11 (0.12) | 39 (16) |
| Malate | 0.74 (0.09) | 2.8 (0.7) | 5.1 (1.5) | 0.073 (0.041) | 11 (6) |
| Succinate | 0.19 (0.05) | 1.7 (0.6) | 1.2 (0.4) | 0.026 (0.018) | 2.6 (1.5) |
| Adenosine monophosphate | 0.098 (0.028) | 2.7 (0.5) | 1.9 (0.6) | 0.28 (0.19) | nq |
| Adenosine diphosphate | 3 (0.2) | 7.6 (1) | 6.3 (1.4) | 0.35 (0.11) | nq |
| Adenosine triphosphate | 38 (2) | 15 (2) | 36 (4) | 0.57 (0.33) | nq |
| Aspartate | 1.4 (0.2) | 4.5 (1.4) | 8.6 (2.3) | 0.24 (0.05) | 5.4 (1.9) |
| Glutamate | 4 (0.7) | 7.3 (3.4) | 27 (3) | 0.52 (0.1) | 20 (2) |
| Histidine | 0.93 (0.07) | 20 (13) | 2 (0.2) | 0.045 (0.029) | 78 (6) |
| Leucine + isoleucine | 0.8 (0.13) | 2.4 (0.6) | 1 (0.2) | 0.16 (0.06) | 140 (20) |
| Lysine | 1.2 (0.2) | 4.9 (1.3) | 2.6 (0.2) | 0.23 (0.16) | 190 (20) |
| Methionine | 0.27 (0.02) | 0.28 (0.07) | 0.42 (0.03) | 0.047 (0.017) | 37 (4) |
| Phenylalanine | 0.28 (0.02) | 0.29 (0.08) | 0.29 (0.04) | 0.042 (0.012) | 33 (3) |
| Proline | 1.2 (0.2) | 0.79 (0.26) | 0.59 (0.05) | 0.078 (0.027) | 72 (11) |
| Threonine | 0.63 (0.07) | 0.62 (0.14) | 1 (0.1) | 0.097 (0.024) | 55 (10) |
| Tyrosine | 0.47 (0.08) | nq | 0.5 (0.06) | 0.065 (0.018) | 62 (13) |
| Valine | 0.47 (0.07) | 2.2 (0.6) | 0.59 (0.08) | 0.093 (0.032) | 100 (20) |
